# Thermal conductivity of skutterudite CoSb_3_ from first principles: Substitution and nanoengineering effects

**DOI:** 10.1038/srep07806

**Published:** 2015-01-22

**Authors:** Ruiqiang Guo, Xinjiang Wang, Baoling Huang

**Affiliations:** 1 Department of Mechanical and Aerospace Engineering, The Hong Kong University of Science and Technology, Clear Water Bay, Kowloon, Hong Kong; 2 The Hong Kong University of Science and Technology Shenzhen Research Institute, Shenzhen, 518057, China

## Abstract

CoSb_3_-based skutterudites are promising intermediate-temperature thermoelectric materials and fundamental understanding of the thermal transport in CoSb_3_ is crucial for further improving its performance. We herein calculate the lattice thermal conductivity *κ_L_* of CoSb_3_ with first-principles methods and conduct a comprehensive analysis on phonon mode contribution, relaxation time and mean free path (MFP) distributions. The contribution of optical phonons is found to be significant (28% at 300 K) and important optical modes usually involve two or more pnicogen atoms moving synchronously. The MFP (~135 nm at 300 K) corresponding to 50% *κ_L_* accumulation in CoSb_3_ is much larger than that predicted from the kinetic theory (~4 nm), providing an opportunity to reduce *κ_L_* by nanoengineering. The effects of elemental substitution and nanoengineering on *κ_L_* are therefore investigated. A 10% substitution of Sb by As results in 57% reduction of *κ_L_* while the in-plane (cross-plane) *κ_L_* of a 50-nm CoSb_3_ thin film is only 56% (33%) of the bulk *κ_L_* at 300 K. The impurity scattering and boundary scattering mainly suppress phonons in different frequency regimes. By combining these two effects, *κ_L_* can be reduced by more than 70% at 300 K, potentially leading to much improved *ZT* near room temperature.

CoSb_3_-based skutterudites are among the most prospective thermoelectric (TE) materials for intermediate-temperature power generation, owing to their high performance, low cost and great potential for tailoring the thermal and electrical transport properties through structural engineering. The efficiency of thermoelectric materials is often measured by a dimensionless figure of merit *ZT* = *S*^*2*^*σT/*(*κ_L_* *+* *κ_e_*), where *S* is the Seebeck coefficient, *σ* the electrical conductivity, *T* the temperature, and *κ_L_* and *κ_e_* are the lattice and electronic contributions to the thermal conductivity *κ*, respectively. CoSb_3_ is a binary skutterudite, which has a general formula MX_3_ (*IM3*) where M is a transition metal atom (Co, Rh, or Ir) and X is a pnicogen atom (P, As or Sb) and is characterized by a cubic crystalline structure containing large cages and four-membered planar rings of X^1^. Binary skutterudites have superior *S*^*2*^*σ* due to their appropriate bandgaps and high carrier mobilities, but their thermal conductivities are also high, impeding their applications as efficient TE materials. A successful strategy to improve *ZT* of skutterudites is through reducing *κ_L_* by filling atoms into lattice cages^2^,^3^,^4^,^5^,^6^,^7^, elemental substitution^8^,^9^,^10^,^11^ or nanoengineering^11^,^12^. Sale's et al.^2^ reported a *ZT* of ~1.0 at 800 K in the filled skutterudite LaFe_3_CoSb_12_ that has a reduced *κ_L_* as low as ~0.9 W/mK due to the “rattling” atoms. Liu et al.^9^ and Su et al.^10^,^11^ synthesized n-type nanostructured CoSb_2.75_Sn_0.05_Te_0.20_ and CoSb_2.75_Ge_0.05_Te_0.20_ compounds in which the elemental substitution and in-situ generated quantum dots reduce *κ_L_* below 1.0 W/mK at ~800 K, leading to *ZTs* as high as 1.1. Qiu et al.^13^ achieved a maximum *ZT* of ~0.7 at 600 K by doping CoSb_3_ with Ga atoms, which occupy both the voids and Sb sites. Toprak et al.^12^ obtained a highest *ZT* ~ 0.17 at 611 K for the pure nanocrystalline CoSb_3_ with average grain size of 220 nm, which has a *κ_L_* ~ 0.8 W/mK because of the enhanced boundary scattering. Although significant reduction of *κ_L_* has been achieved in these materials, the underlying mechanism is still not fully understood. For example, the average phonon mean free path (MFP) predicted from the kinetic theory is only ~4 nm, which cannot explain the substantially reduced *κ_L_* in the nanocrystalline CoSb_3_ with average grain size of a few hundred nanometers^12^. Also, the reported *κ_L_* values are still much larger than the theoretical alloy limit ~0.3 W/mK^14^. The open question is how *κ_L_* of skutterudites can be further reduced and by how much. The key to answer these questions is a rigorous prediction of *κ_L_* and a detailed knowledge of intrinsic phonon transport in CoSb_3_. To reduce *κ_L_* through structural engineering, phonon MFP distribution and spectral contributions to *κ_L_* is most important. Unfortunately, to date, accurate atomic-scale investigations on these details in CoSb_3_ are still rare.

Lattice dynamics in bulk CoSb_3_ have been intensively investigated. Lutz et al.^15^ and Nolas et al.^16^,^17^ investigated the lattice dynamics at the Γ point using infrared and Raman spectroscopy, respectively. Rotter et al.^18^ measured the phonon dispersion along [001] direction by inelastic x-ray scattering experiments. First-principles calculations^18^,^19^,^20^ have been conducted to calculate the phonon dispersion and density of states (DOS), which show good agreements with the experimental results. A few classical force constant models have also been proposed by Lutz et al.^15^ and Feldman et al.^21^ through fitting with the first principles calculations and experimental phonon frequencies. These lattice dynamics investigations pave the way for the prediction of *κ_L_*. Analytical phonon conductivity models^1^ based on Boltzmann transport equation (BTE), typically in Callaway-Holland form, have been developed to understand the effects of defects in bulk skutterudites. *κ_L_* of skutterudites can also be reasonably evaluated with Slack's model^22^. However, these models heavily rely on the fitting parameters and might blur the underlying heat transfer physics. Recently, Li et al.^23^ investigated the effects of fillers on *κ_L_* of fully-filled skutterudites by analyzing phonon relaxation times and phonon dispersion modifications obtained from the ab initio calculations. However, a comprehensive understanding of the mode-wise phonon transport in CoSb_3_ is still needed, which is useful for reducing *κ_L_* through selective doping or nanoengineering.

In this study, we accurately determine *κ_L_* of CoSb_3_ by using first principles and BTE. Detailed analysis on the mode-wise phonon relaxation time, MFP and spectral contributions to *κ_L_* has been conducted. It is found that optical phonons contribute significantly to *κ_L_* and the MFP (~135 nm at 300 K) corresponding to 50% *κ_L_* accumulation is surprisingly large, implying great potential for reducing *κ_L_* through nanoengineering. The effects of impurity scattering caused by element substitution and boundary scattering on spectral phonon transport are clarified. A microscopic understanding about the vibration behavior of the pnicogen rings and the effects of substitution is given. While the impurity scattering mainly affects optical phonons, the diffusive boundary can efficiently suppress acoustic phonon transport. It is shown that combining these two scattering mechanisms can significantly decrease *κ_L_*. The improvement of *ZT* benefited from *κ_L_* reduction may notably broaden the application temperature range of CoSb_3_-based skutterudites.

## Results

**Figure 1:**
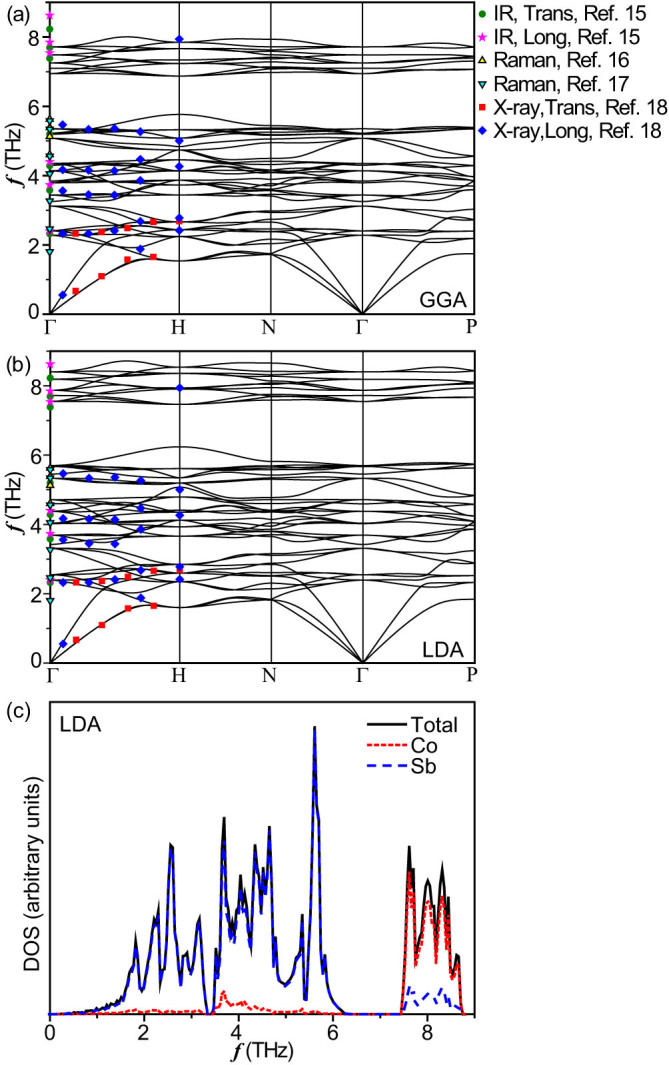
Phonon dispersion along different high-symmetry paths for GGA (a) and LDA (b) calculations. Experimental results from Refs 15,16,17,18 are denoted by filled symbols. (c) Total and partial phonon density of states for CoSb_3_.

*κ_L_* is determined by both the harmonic and anharmonic interatomic interactions and it is essential to understand the lattice dynamics in CoSb_3_ and the corresponding phonon distribution. The interaction strength between atoms from ab-initio calculations relies on the approximations to exchange and correlation, which will affect the lattice parameters and the forces on atoms used for extracting the phonon dispersion. We adopted the local density approximation (LDA) and generalized gradient approximation (GGA) treatments to optimize the CoSb_3_ structure, yielding a lattice constant of 8.92 Å and 9.11 Å, respectively. Compared with the experimental value *a* = 9.04 Å^24^, the LDA underestimates *a* by 1.3% while the GGA overestimates it by 1.1%, agreeing well with previous ab-initio predictions^18^,^20^,^25^ and following the well-known overbinding and underbinding tendency of the LDA and GGA. Figures 1(a) and (b) show the phonon dispersions of bulk CoSb_3_ for LDA and GGA calculations along some high-symmetry directions, in comparison with the experimental results^15^,^16^,^17^,^18^. Overall both LDA and GGA reproduce well the acoustic branches while for optical phonons the LDA calculations show better agreements with the experimental values. Therefore, the LDA can be taken as a reliable base for the subsequent calculations. The acoustic group velocities are found to be 2601 m/s for TA1 (transversal acoustic), 2881 m/s for TA2 and 4716 m/s for LA (longitudinal acoustic) respectively, agreeing with the experimental TA of 2643 m/s and LA of 4590 m/s^26^. It is known that the elastic constants are directly related to the acoustic group velocities. Our calculations yield the elastic constants *C*_11_ 172.0 GPa, *C*_12_ 43.7 GPa and *C*_44_ 57.7 GPa, respectively, which are consistent with the experimental results^27^. Figure 1(c) shows the total phonon DOS of CoSb_3_ characterized by a large band gap between 6.3 and 7.4 THz. The band gap is related to the mass mismatches of constituent atoms. It has been shown that the band gap increases with increasing the cation/anion mass ratio in binary wurtzite nitrides^28^. The partial DOS of CoSb_3_ is also shown in Fig. 1(c) and significantly different vibration properties of Co and Sb atoms are illustrated. The vibration of Co atoms dominates the high frequency spectrum above 7.4 THz while the low frequency spectrum below 6.3 THz mainly comes from the vibration of Sb atoms. Such vibrational properties provide an opportunity to selectively tune the phonon spectrum in separate frequency regime by substituting different atoms in CoSb_3_.

**Figure 2:**
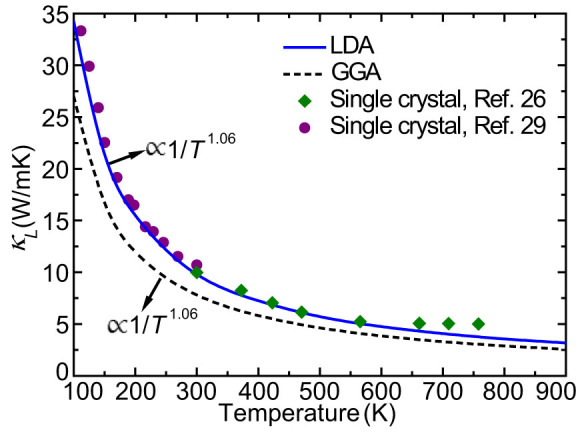
*κ_L_* of CoSb_3_ for LDA and GGA with respect to temperature, compared with the experimental results (denoted by filled symbols) from Refs 26, 29.

*κ_L_* of bulk CoSb_3_ for LDA and GGA as a function of temperature are shown in Fig. 2, in comparison with the experimental results^26^,^29^. From 100 K to 900 K, the LDA predictions are relatively larger than the GGA values and *κ_L_* shows an approximate 1/*T* temperature dependence for both LDA and GGA, a common behavior for crystals. It is found that the LDA calculations match better with the experimental results while GGA tends to underestimate *κ_L_*. For example, the LDA *κ_L_* of 9.8 W/mK at 300 K is very close to the experimental value 10 W/mK of single crystals while the GGA produces a lower *κ_L_* of 7.8 W/mK. The latter agrees well with the result from Huang et al.^20^ obtained by using classical molecular dynamics simulations, in which the potentials were fitted from first-principles calculations with GGA. The underestimation by GGA calculations is mainly caused by the underbinding behavior of GGA, which makes the phonon dispersion shift to lower frequencies and results in slightly lower group velocities. The average acoustic group velocity for GGA is ~8.7% smaller than that for LDA, which can lead to a thermal conductivity ~17% lower according to the kinetic theory 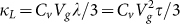 (*C_v_*, *Vg*, *λ* and *τ* represent the phonon volumetric specific heat, group velocity, MFP and relaxation time, respectively). Above 700 K, the calculated *κ_L_* are slightly lower than the experimental results, for which the electronic contributions (for example, ~3% at 700 K based on Wiedemann-Franz law) to the total thermal conductivity have been removed. This deviation might be caused by bipolar heat conduction at high temperatures. The effects of phonon-isotope scattering on *κ_L_* is insignificant for CoSb_3_ in the temperature range considered. Natural isotopes result in a largest reduction (4%) of *κ_L_* at 100 K with respect to the isotopically pure materials. Li et al.^23^ obtained a larger *κ_L_* of 11.5 W/mK at 300 K using a similar method, which may be caused by the relatively small cutoff of 4 Å adopted for the interaction range. Actually, the long-ranged interactions may be important for the phonon transport, which has been highlighted in previous investigations^30^. We found that the harmonic interatomic force constants almost vanish at a cutoff interatomic distance of 6.5 Å (See the Supplementary Information).

**Figure 3:**
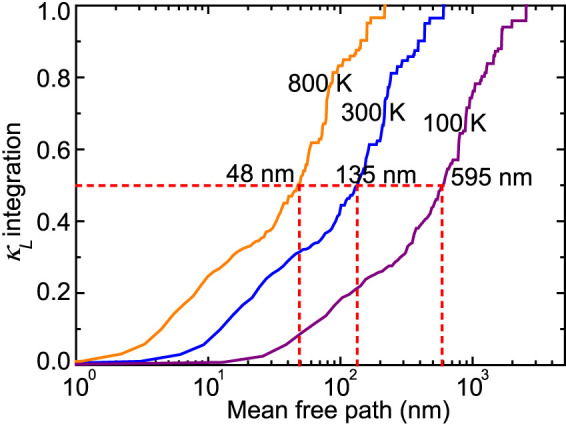
Normalized *κ_L_* accumulation for CoSb_3_ at 100, 300 and 800 K, as a function of phonon mean free path.

The average MFP can be predicted according to the kinetic theory, yielding a very short average MFP of ~4 nm at 300 K. Considering the uneven contribution of different modes, this gray approximation might be misleading, as for MFP found in Si^31^. The knowledge of the contribution of different phonon modes to *κ_L_* is helpful for understanding the intrinsic phonon transport and can provide guidance for the structural engineering. Figure 3 shows the normalized *κ_L_* accumulation with respect to the MFP of bulk CoSb_3_ at 100, 300 and 800 K, respectively. The MFPs corresponding to 50% *κ_L_* accumulation at 100, 300 and 800 K are respectively 595, 135 and 48 nm, which, however, are surprisingly long. For example, the accumulation median MFP value for CoSb_3_ approaches ~40% of the corresponding one of bulk Si (~350 nm) at 300 K although *κ_L_* of Si (155 W/mK at 300 K^32^) is more than one order of magnitude higher. This difference can be simply explained by kinetic theory. When only acoustic phonons (contribute ~70% to the overall *κ_L_* in CoSb_3_ at 300 K) are considered, the thermal conductivity ratio *κ_L_*(Si)/*κ_L_*(CoSb_3_) ≈ [*C_v_*(Si)*v_g_*(Si)*λ*(Si)]/[*C_v_*(CoSb_3_)*v_g_*(CoSb_3_)*λ*(CoSb_3_)] ≈ 22.5, reasonably agreeing with the ab initio predictions. Here, the ratios of *C_v_* (estimated by the number density of unit cell through the Dulong-Petit law^33^), *v_g_* (that of Si is ~6300 m/s^34^) and *λ* are about 4.5, 2 and 2.5, respectively. The relatively large phonon MFPs in CoSb_3_ rationalize the reported experimental results^12^ and suggest the potential to decrease *κ_L_* by nanoengineering. It is also noted that the phonon modes contribute unevenly to the thermal conductivity. For example, at 300 K, the phonon modes with MFPs shorter than 100 nm contribute 42% to *κ_L_* while those with MFPs longer than 400 nm only contribute 10% to *κ_L_*. Consequently, the gray approximation for MFP may lead to a large uncertainty in the analysis. This further confirms the necessity of using mode-dependent parameters for the modeling of *κ_L_*. At 800 K, the 50% thermal conductivity accumulation corresponds to a MFP of 48 nm, which is relatively challenging to be further reduced by nanoengineering.

**Figure 4:**
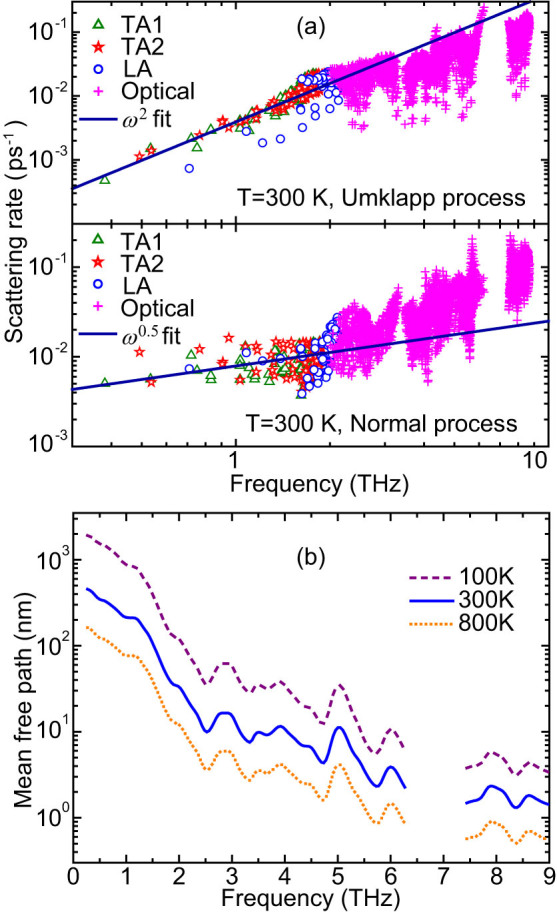
Phonon scattering rates for Umklapp process and Normal process at 300 K (a) and average mean free path (b) at 100, 300 and 800 K with respect to frequency for CoSb_3_.

Figure 4(a) further shows the phonon scattering rates for the Umklapp and Normal processes with respect to frequency at 300 K, which are obtained from the single-mode relaxation time approximation (SMRTA). The sum of the Normal and Umklapp scattering rates would be the inverse of the relaxation time, for which SMRTA and iterative method yield very close values and the deviation is less than 1%. The scattering rates of three acoustic branches and optical modes are plotted separately. It can be found that for both processes the scattering rates generally increase with the increase of frequency, indicating the enhanced phonon scattering. Callaway^35^ suggested a *ω*^2^ dependence of scattering rates for the Umklapp process, which is often used to predict *κ_L_*. Here, we found a quadratic dependence of the Umklapp scattering rates for acoustic phonons on frequency while Normal scattering rates are proportional to ~*ω*^0.5^ at 300 K. It is also noted that at low frequencies (typically < 1.5 THz) the Normal process dominates while the scattering rates of the two processes are comparable at higher frequencies (typically for optical modes). Due to different intrinsic scattering channels in materials, the frequency dependence of scattering rates may be different. For example, it has been reported that the acoustic scattering rates due to Normal and Umklapp processes at 277 K in bulk Si are proportional to *ω*^2^ and *ω*^3^ respectively^31^. Figure 4(b) shows the mode-averaged MFP distributions with respect to frequency at 100, 300 and 800 K, respectively. Typically, the MFP decreases with the increase of temperature due to the enhanced phonon scattering. Because of the reduction of the relaxation time and group velocity, the MFP will be suppressed when the frequency increases. From Fig. 4(b), one can find that acoustic phonons generally possess a relatively long MFP. At 300 K, the MFPs of acoustic phonons increase from 20 nm to ~500 nm below 2.2 THz while most optical phonons (*f* > 2.2 THz) possess a MFP shorter than 10 nm. Therefore, nanoengineering will significantly suppress acoustic phonons while optical phonons will be less affected. Even at 800 K, the MFPs of most acoustic phonons are above tens of nanometers while optical phonons typically have a MFP shorter than 5 nm.

**Figure 5:**
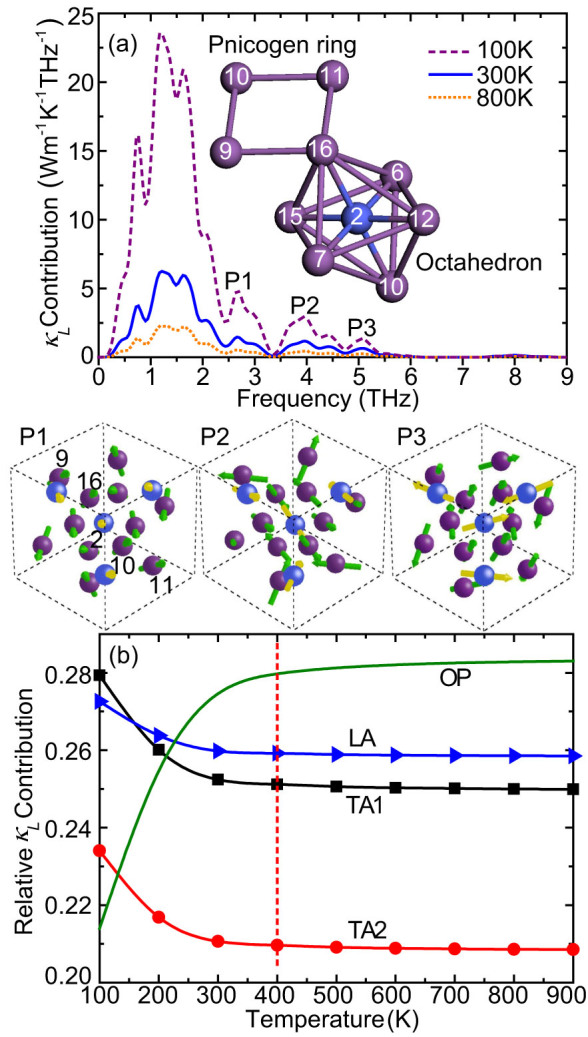
(a) *κ_L_* contribution with respect to frequency at 100, 300 and 800 K for CoSb_3_. The optical vibration modes corresponding to the peaks P1, P2 and P3 are shown in the bottom (eigenvectors are indicated by arrows). Inset shows a typical skutterudite structure consisting of the pnicogen ring and octahedron. Co and Sb atoms are colored by blue and purple, respectively. (b) Relative *κ_L_* contribution of TA1, TA2, LA and optical (OP) phonon branches with respect to temperature.

**Figure 6:**
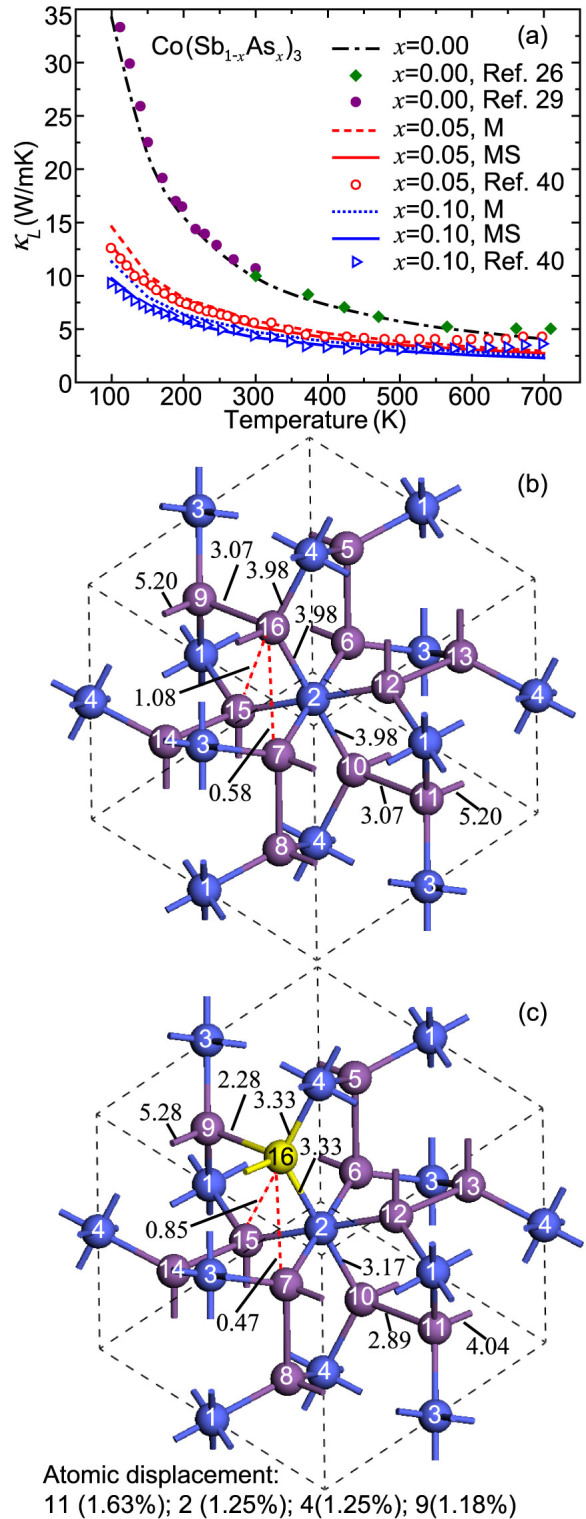
(a) Temperature-dependent *κ_L_* of Co(Sb_1-*x*_As*_x_*)_3_. The ab-initio and experimental results from Refs 26, 29 (single crystal) and 40 (polycrystal) are represented by lines and symbols, respectively. “M” means only the mass variance is considered while “MS” incorporates the effects of both mass variance and strain field fluctuation. Comparisons between the longitudinal force constants of Co_4_Sb_12_ (b) and Co_4_Sb_11_As_1_ (c) obtained from first principles are also shown. Co atoms 1–4 and Sb atoms 5–16 are colored by blue and purple, respectively. The Sb atom 16 is substituted by As and represented by yellow color.

Figure 5(a) shows *κ_L_* contribution with respect to frequency at 100, 300 and 800 K, respectively. The curves for different temperatures have a similar shape with a peak position at ~1.2 THz. The acoustic phonons between 0.5 and 2.0 THz dominate *κ_L_* contribution. Despite the dominance of acoustic phonons, the optical phonons still contribute ~27.7% of the total *κ_L_* at 300 K. At 800 K, the relative contribution of optical phonons slightly increases to 28.3%. However, the optical phonons with frequency >5.5 THz contribute little to *κ_L_* at all these temperatures. Significance of optical phonons has also been highlighted in some previous works^36^,^37^,^38^. In materials such as Si nanowires^37^, PbSe^38^ and PbTe^38^, optical phonons contribute ~20% to *κ_L_*. One can find that there are three peaks P1, P2 and P3 in the optical frequency regime, which result from some important phonon modes with relative large *κ_L_* contributions. The corresponding vibration modes are shown in the bottom panel of Fig. 5(a). The skutterudites contain two typical substructures: the pnicogen ring (Sb4 ring for CoSb_3_) and the octahedron, as shown in the inset of Fig. 5(a). It is of great importance to observe the vibration of the pnicogen rings because dominant heat-carrying modes in skutterudites are related with their vibrations^8^,^10^,^11^. Due to the covalent characteristic, the bonds of Sb4 rings are relatively strong. The force constants obtained from first principles also support this (Fig. 6). The vibration modes are shown in a typical primitive cell, where the middle Co atom is surrounded by 6 first-neighbor Sb atoms that form an octahedron. Based on the space symmetry, one can find that the diagonal four Sb atoms 9, 10, 11 and 16 would form a Sb4 ring, as shown in the inset of Fig 5(a). It is found that the diagonal atoms on the Sb4 ring have parallel eigenvectors but may vibrate out of sync. For the mode P1, the rings exhibit a translational-like motion with all ring atoms vibrate almost synchronously. For the mode P2, the atoms on each long Sb-Sb bond move together with the bond length slightly changed during the vibration while the two long bonds (16-9 and 11-10) vibrate in the opposite direction. The mode P3 has a similar vibration behavior for the Sb4 ring except that the atoms on the short Sb-Sb bonds are bonded and move together. Generally, the six Sb atoms in the octahedron vibrate asynchronously and along different directions, which leads to significant deformation for the octahedron. From these typical modes, it can be found that the atoms on the pnicogen rings seldom move randomly; instead, two or more pnicogen atoms are usually bonded together, which can be attributed to their strong covalent bonds. Therefore, if this bonding behavior is disturbed *κ_L_* contribution of related phonon modes may be significantly reduced. This provides a microscopic understanding about *κ_L_* suppression caused by the substitution of pnicogen atoms^8^,^10^,^11^. Specifically, Chi et al.^8^ found that double substitution is an effective way to disturb the pnicogen rings.

It is informative to examine *κ_L_* contribution from different phonon branches, as shown in Fig. 5(b). The contribution of optical phonons is plotted by summing up over all optical phonon modes. Due to the enhanced Umklapp scattering, the contribution of acoustic phonons decreases as the temperature increases. The contributions from the three acoustic branches are comparable and the longitudinal branch contributes the most above 160 K, probably due to its large group velocity. When temperature increases, owing to the fast reduction in the contribution of acoustic phonons, the relative contributions from optical phonons increase although their absolute contributions to *κ_L_* decrease because of the enhanced scattering, as shown in Fig. 5(a). Overall, the relative contributions of different branches vary little with temperature and almost keep constant above 400 K.

The phonon contribution analysis points out the possible directions to tune *κ_L_* of CoSb_3_. Considering the relative large MFPs of acoustic phonons, nanoengineering will be effective in reducing *κ_L_* by boundary scattering. Also, the effect of impurity scattering on *κ_L_* should be notable because of the significant contribution from optical phonons. Thus, it is expected that a combination of these two mechanisms will greatly suppress *κ_L_* of CoSb_3_. Next, we'll examine how much *κ_L_* can be reduced by these two scatterings and analyze the underlying mechanisms. Impurity scattering can be generated in CoSb_3_ by two approaches: filling atoms into the lattice cages and elemental substitution. Filled CoSb_3_ structures have been extensively studied^2^,^20^,^39^. In fact, there are more elements available for the substitution of Co and Sb elements, and most processes can be used for the synthesis. According to the DOS, it is known that Sb atoms dominate the low-frequency phonon spectrum. More importantly, the substitution of the Sb atom will significantly disturb the pnicogen rings, which play an important pole in the thermal transport of skutterudites. Therefore, we herein introduced impurity scattering by substituting Sb atoms with As atoms.

*κ_L_* of Co(Sb_1-*x*_As*_x_*)_3_ with different As concentrations as a function of temperature were calculated by including both effects of mass variance and strain field fluctuation, in comparison with those calculated by only considering mass variance and experimental results^26^,^29^,^40^, as shown in Fig. 6(a). It is found that the calculated results for both cases show a similar tendency to the experimental values. The substitution of Sb can significantly lower *κ_L_* of CoSb_3_, e.g., a 10% substitution of Sb element with As results in a 57.1% reduction of *κ_L_*. The further reduction of *κ_L_* caused by strain field scattering is significant below 200 K but gradually decreases as the temperature increases. This is because the relative contribution of strain filed scattering becomes smaller due to the enhanced phonon scattering at higher temperatures. Our calculations indicate that the scattering due to mass variance is much larger than strain field scattering, which is different from the previous estimations based on adjustable parameters^40^. It is also noted that the experimental results are higher than the calculated values above 600 K. The electronic contribution is found to be less than 4% to the total thermal conductivity, implying that bipolar heat conduction may be important at high temperatures. The strain field scattering originates from the modification of atom position and interatomic coupling force^40^,^41^. To confirm this, we calculated the atomic displacements and the longitudinal force constants *Γ*_L_ between various atoms in the substituted and un-substituted structures, as shown in Figs. 6(b) and (c). It is found that the atomic displacements for the four neighbor atoms of As (>1.1%) are significantly larger than those of other atoms (<0.6%). In CoSb_3_, the largest three *Γ*_L_ come from the Co-Sb bond (3.98 eV/Å^2^, 16-4) in the octahedron and the short (5.20 eV/Å^2^, 16-11) and long (3.07 eV/Å^2^, 16-9) Sb-Sb bonds in the Sb4 ring. The substitution of Sb atom 16 by As atom has a significant affection on the surrounding bonds. Most *Γ*_L_ between the atom 16 and other atoms are reduced, indicating that the bonds are softened. For example, the *Γ*_L_ 16-9, 16-11 and 16-4 are reduced by 25.8%, 22.4% and 16.3% respectively. Similar bond softening was also reported in other studies on skutterudites with filled atoms^20^.

**Figure 7:**
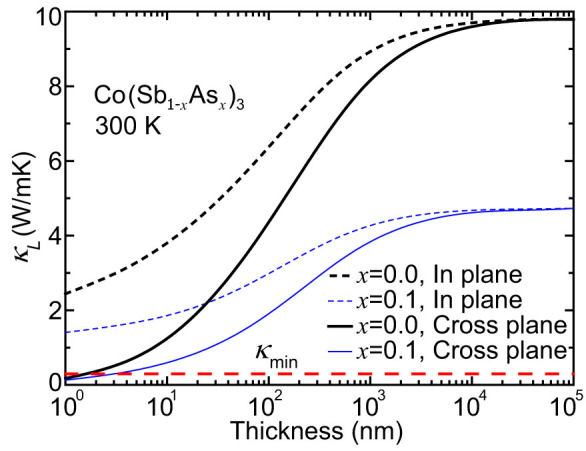
Thickness dependence of *κ_L_* in Co(Sb_1-*x*_As*_x_*)_3_ for *x* = 0.0 and 0.1. The theoretical minimum *κ*_min_ is also shown.

To investigate the effect of boundary scattering on thermal conductivity of Co(Sb_1-*x*_As*_x_*)_3_, we obtained the thickness dependence of the in-plane and cross-plane *κ_L_* of Co(Sb_1-*x*_As*_x_*)_3_ films at 300 K, as shown in Fig. 7. For both structures (*x* = 0.0 and 0.1), boundary scattering begins to markedly affect *κ_L_* when the thickness is smaller than ~1 *μ*m. If the thickness of un-substituted CoSb_3_ decreases to 50 nm, its in-plane (cross-plane) *κ_L_* can be reduced by 44.1% (67.4%). It is expected that boundary scattering will have a stronger influence on *κ_L_* of the substituted structures, in which the contribution of low-frequency phonons should be relatively larger due to the impurity scattering. For Co(Sb_0.9_As_0.1_)_3_ thin film with a thickness of 50 nm, its in-plane (cross-plane) *κ_L_* is reduced by 73.9% (85.9%) with respect to the bulk value of CoSb_3_. The thickness corresponding to 50% reduction of in-plane *κ_L_* in the substituted one is 34 nm. It is inspiring to find that combining these two scatterings can result in a greatly reduced *κ_L_*. For example, the in-plane (cross-plane) *κ_L_* of Co(Sb_0.9_As_0.1_)_3_ with a thickness of 50 nm is only 2.6 (1.4) W/mK at 300 K, much lower than 9.8 W/mK for bulk CoSb_3_. It has been experimentally found that the power factor of bulk Co(Sb_0.9_As_0.1_)_3_ increases by 176% with respect to that of the CoSb_3_^40^. Considering the short electronic MFP, it is reasonable to assume a little-deteriorated power factor in corresponding thin films. As a result, *ZT* of bulk CoSb_3_ can be potentially improved by about 10 (19) times at room temperature.

## Discussion

**Figure 8:**
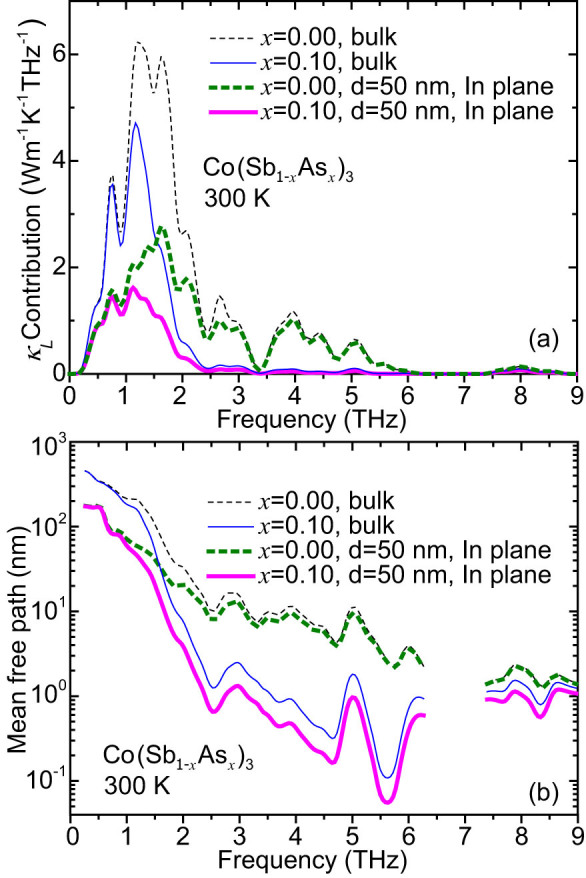
*κ_L_* contribution (a) and phonon mean free path (b) with respect to frequency for bulk Co(Sb_1-*x*_As*_x_*)_3_ and thin films of a thickness of 50 nm with different As concentrations.

To further understand the influence of impurity scattering and boundary scattering, we calculated *κ_L_* contribution with respect to frequency and MFP distribution for different structures, as shown in Figs. 8(a) and (b). From Fig. 8(a), one can find that the substitution mainly affects the phonons with frequency >1 THz, the contributions of which to *κ_L_* are greatly reduced. But acoustic phonons with frequency <1 THz are almost unaffected. The reduction of film thickness also has an impact on both acoustic and optical phonons. When the thickness of CoSb_3_ reduces to 50 nm, the in-plane contributions of acoustic and optical phonons are reduced by 54.2% and 14.7% respectively. For the Co(Sb_0.9_As_0.1_)_3_ thin film with the same thickness, compared with its bulk, the boundary scattering reduces the in-plane contributions of acoustic and optical phonons by 57.5% and 42.3% respectively. For both thin films, the further reduction of *κ_L_* caused by boundary scattering is mainly due to the suppression of low-frequency phonons. Figure 8(b) shows the MFP distribution with respect to phonon frequency at 300 K. It is noted that the phonon MFPs between 1 and 6 THz are mainly decreased due to the substitution. When the substitution fraction increases, the MFPs of these phonons are further reduced. But the MFPs of acoustic phonons with frequency <1 THz are hardly affected. The modification of MFP caused by boundary scattering is very different. For the CoSb_3_ thin film with a thickness of 50 nm, the MFPs of most acoustic phonons are reduced by more than 50% while the MFPs of high-frequency phonons (>3 THz) almost remain the same. For the Co(Sb_0.9_As_0.1_)_3_ thin film, however, the MFPs of optical phonons from 2.2 to 6 THz are also remarkably decreased by ~50% with respect to its bulk, accounting for the corresponding significant reduction of *κ_L_*.

In summary, the intrinsic *κ_L_* of CoSb_3_ has been accurately predicted using BTE with the harmonic and anharmonic parameters obtained from first-principles calculations. A comprehensive analysis about phonon mode contribution, relaxation time and MFP distributions was conducted. Optical phonons contribute significantly to *κ_L_* (28% at 300 K) and their relative contributions slightly increase with temperature. To reduce *κ_L_*, the suppression of optical phonons should be considered. We also found that the MFP (~135 nm at 300 K) corresponding to 50% *κ_L_* accumulation in CoSb_3_ is much larger than that predicted from the kinetic theory (~4 nm), providing an opportunity to reduce *κ_L_* by nanoengineering. Interestingly, the vibrations of some important optical modes show that two or more pnicogen ring atoms usually move synchronously due to their strong covalent bonds. This provides a microscopic understanding about *κ_L_* reduction caused by the substitution of pnicogen atoms.

Accordingly, we investigated the effects of impurity scattering generated by elemental substitution and boundary scattering in thin films on *κ_L_*. To accurately predict *κ_L_* of the substituted structures, both mass variance and strain field fluctuation should be considered due to the significant atomic displacements and bond softening. It is found that a 10% substitution of Sb element with As results in a 57.1% reduction of *κ_L_* while the in-plane (cross-plane) *κ_L_* of a 50 nm CoSb_3_ thin film is only 55.9% (32.6%) of the bulk value at 300 K. By combining these two effects, *κ_L_* can be reduced by 73.9% (85.9%). Further analysis shows that the substitution mainly suppresses the phonons with frequency >1 THz. In thin films of Co(Sb_1-*x*_As*_x_*)_3_, the reduction of *κ_L_* is mainly caused by the great reduction in MFPs of acoustic phonons. In this work, we demonstrate that elemental substitution combined with boundary scattering is an effective approach for reducing *κ_L_* of CoSb_3_. The calculated phonon transport details provide guidance for the further reduction of *κ_L_*, which can be achieved if multiple scattering mechanisms are reasonably manipulated. The enhancement of *ZT* benefited from the substantially reduced *κ_L_* may significantly enlarge the efficient working temperature range of CoSb_3_-based thermoelectrics.

## Methods

The lattice thermal conductivity can be predicted based on phonon BTE with harmonic and anharmonic interatomic force constants (IFCs) as inputs, which are determined from ab initio calculations. Recently, many other materials^31^,^38^,^42^,^43^,^44^,^45^,^46^ have been successfully predicted by this method, which show good agreements with the experimental results.

### BTE

At equilibrium state, the phonon distribution is determined by the Bose-Einstein distribution function 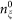, where *ξ*(*q*, *j*) represents a phonon mode denoted by the wavevector *q* and the phonon dispersion branch *j*. By applying a small temperature gradient ∇*T* to disturb the phonon distribution function 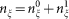, the BTE can be written as
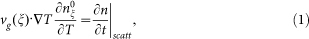
where the nonequilibrium part 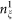 is proportional to the small ∇*T* and v*_g_*(*ξ*) is the group velocity. The left-hand side represents phonon diffusion induced by the temperature gradient and the right-hand side represents the phonon scattering rate due to all scattering process. Matthiessen's rule is often adopted to sum up the effects of independent multiple scattering mechanisms. This study only considers three major scattering mechanisms in CoSb_3_, i.e., intrinsic phonon-phonon scattering, impurity scattering and boundary scattering.

The phonon-phonon scattering relaxation time *τ_p_*_-*p*_ can be calculated by anharmonic lattice dynamics calculations. We herein only consider the three-phonon scattering process that is dominant below 1000 K although it is possible to consider the fourth-order and even higher order anharmonic effects in the calculations, which might play a role at very high temperatures. Three-phonon scattering rates computed from Fermi's golden rule is related to the third-order anharmonic interactions^42^ through
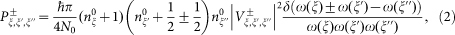
where *ω* is phonon frequency. In this process, the conservation of energy and momentum should be satisfied, i.e., *ω*(*ξ*) ± *ω*(*ξ*′) = *ω*(*ξ*″) and *q*″ + *q* + *q*′ = *G*, where *G* is either 0 for the Normal process or a nonzero reciprocal lattice vector for the Umklapp process. The interaction strength 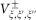 among three phonon eigenmodes *ξ*, *ξ*′ and *ξ*″ can be determined by
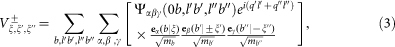
where ψ*_αβγ_*(0*b*,/'*b*',/”*b*”) represents the third-order force constants in terms of atoms *b*, *b'* and *b''* in lattice *0*, *l′* and *l″* of a crystal consisting of *N_0_* cell.

The isotopic impurity scattering rate can be calculated by^47^
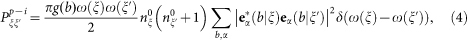
where the mass variance is often characterized as an average parameter
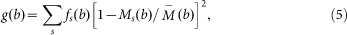
where *f_s_*(*b*) and *M_s_*(*b*) are the concentration and mass of the *s*th isotope of atom *b*, respectively, 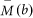 is the average mass of the *b*th atom in the unit cell. Naturally, Co is composed of one stable isotope^59^ Co while Sb consists of ^121^Sb (57.36%) and ^123^Sb (42.64%).

For the case of substitution, the substitutional atom differs from the host atom not only in its mass, but also in its size and the coupling force to its neighbor atoms, which will result in the strain field scattering. Instead of *g*(*b*), a convenient scattering parameter *a_s_*, including both the effects of mass variance and strain field fluctuation, is defined as^8^,^41^
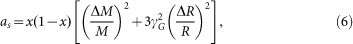
where *x* is the atomic fraction of the impurity atoms, Δ*M* and Δ*R* are the atomic mass and radius difference between the impurity and host atoms, *γ*_G_ is the Grüneisen constant, and *M* and *R* are the average atomic mass and radius. The first term about mass corresponds to the mass variance in Eq. (5). The mode Grüneisen constants *γ_i_*, which provide an estimate of the anharmonicity of the bonds, were directly calculated from first principles. An average *γ*_G_ was obtained for phonons contributing to the thermal transport according to *γ_G_* = ∑*_i_γ_i_*C*_v,i_*/∑*_i_*C*_v,i_*^48^.

Assuming the coordinate *z* is along the cross-plane direction of thin films, the boundary scattering rate can be quantified as
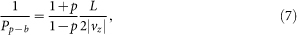
where *L*/2|*V_z_*| represents the average time duration for a phonon to transport from one boundary to the other, provided evenly distributed possibility of emergent angle after scattering. The specularity parameter *p*, ranging from 0 to 1, is used to account for the possibility of specular reflection on the surface. In this work, *p* is set to 0, i.e., a complete diffusive scattering on the boundary is assumed. Therefore, the calculations will lead to a lower bound of *κ_L_* for thin films. For the calculations of in-plane and cross-plane thermal conductivity, the *x* and *z* components of group velocity are adopted respectively.

Equation (1) is solved using an iterative approach^49^,^50^,^51^ to obtain the relaxation time *τ*(*ξ*), which is used to calculate the lattice thermal conductivity
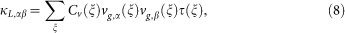
where *κ_L_* is a second-order tensor with the subscripts *α* and *β* denoting its components. *C_v_* is related to the mode frequency by
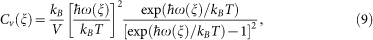
where *k_B_* is the Boltzmann constant and *V* is the volume of the unit cell. *C_v_* and *v_g_* = *∂ω*/*∂q* can be determined by the phonon dispersion relations obtained from harmonic lattice dynamics calculations^52^. To analyze the scatterings caused by the Normal and Umklapp processes, we also calculated *τ*(*ξ*) by adopting the single-mode relaxation time approximation. The relaxation times obtained in these two different methods are very close and the deviation is less than 1%.

The phonon dispersion relations of a crystal can be obtained by solving the eigenvalue problem through diagonalizing the Fourier-transformed harmonic interatomic force constants (dynamical matrix)^33^, as shown in equations (10) and (11)
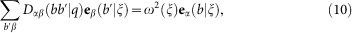

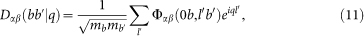
where **e***_a_*(*b*|*ξ*) is the *α* component of the vibration eigenvector for the atom *b* involved in the phonon mode *ξ* and *D_αβ_*(*bb′*|*q*) is the component of the dynamical matrix. Φ*αβ*(0*b*,/'*b*') is the real-space force constant between the atom *b* in the origin unit cell and the atom *b′* in the *l′*th unit cell in the system.

The zeroth-order solution of iterative process is equivalent to the SMRTA, which has been widely adopted for its easier implementation and its explicit relationship with phonon mean free path^45^,^53^. Details about SMRTA have been introduced in our previous work^54^. For many bulk materials^45^,^53^, the predictions by SMRTA are close to those from iterative approach. Here, compared with the results from the iterative method, SMRTA leads to almost the same results for *κ_L_* and relaxation times of CoSb_3_ and the deviations are generally less than 1% within the temperature range interested, Based on SMRTA, the Normal and Umklapp scattering rates can be obtained, respectively.

### Ab initio calculation of IFCs

The harmonic and third-order anharmonic IFCs were obtained from first-principles calculations, which were implemented with the projector augmented wave (PAW)^55^ pseudopotentials, LDA of Perdew and Zunger^56^ and the Perdew-Burke-Ernzerhof (PBE)^57^ form of GGA using the Vienna Ab initio simulation package^58^ (VASP). A conventional unit cell of CoSb_3_ with 32 atoms was first relaxed with a 11 × 11 × 11 Monkhorst-Pack **k** sampling grid and a cut-off energy of 400 eV. The IFCs were then calculated in the real space using the frozen-phonon method^59^. A 2 × 2 × 2 supercell and a conventional unit cell were used for second-order and third-order IFCs calculations, respectively. The static first-principles calculations were conducted with a precision as high as 10^−8^ eV for the total energy difference between two self-consistency steps and 4 × 4 × 4 **k**-points to obtain the forces on each atom within the perturbed systems. The convergence criterion for the forces on atoms was set to 10^−6^ eV/Å. After the extraction of the harmonic and anharmonic IFCs, the frequency and relaxation time of each mode were calculated by conducting a Fourier transformation with a dense **q** mesh (reciprocal space mesh for phonons) scheme 11 × 11 × 11, corresponding to a 11 × 11 × 11 supercell in the real space, which has been tested to be enough to yield convergent results in the temperature range from 100 K to 900 K. Further increase in the mesh scale has little effect on the predicted thermal conductivity values.

## Author Contributions

R.G. carried out the ab-initio calculations and data analysis and prepared all the figures. X.W. developed the code. R.G. and B.H. wrote the manuscript. All authors have reviewed, discussed and approved the results and conclusions of this article.

## Supplementary Material

Supplementary Information Supplemental Informaiton
