# Supplementary material for: Thermal conductivity of skutterudite CoSb3 from first principles: Substitution and nanoengineering effects
Source: Sci Rep. 2015 Jan 22;5:7806. doi: 10.1038/srep07806 (PMC4302311; doi:10.1038/srep07806)
Supplement: Supplementary Information — Supplemental Informaiton [file srep07806-s1.pdf]

Supplementary Information

Thermal conductivity of skutterudite $CoSb_3$ from first principles: Substitution and nanoengineering effects

Ruiqiang Guo[1], Xinjiang Wang[1] and Baoling Huang[1,2*]

[1]Department of Mechanical and Aerospace Engineering, The Hong Kong University of Science and Technology, Clear Water Bay, Kowloon, Hong Kong

[2]The Hong Kong University of Science and Technology Shenzhen Research Institute, Shenzhen, 518057, China

* Correspondence and requests for materials should be addressed to B.L.H. (mebhuang@ust.hk)

I. Determination of the cutoff interatomic distance

Figure 1 shows the harmonic interatomic force constants (IFCs) for $CoSb_3$ normalized by the self-interacting IFC, which means the IFC of one specific atom when the atom itself is displaced. The same treatment has been adopted in Lee et al.'s work[1]. The lattice thermal conductivity is directly related to IFCs. For economic computations, one can typically choose a cutoff interatomic distance, above which the IFCs are negligible. The cutoff distance should be carefully determined because the long-ranged interactions may be important for the phonon transport, which has been highlighted in Lee et al.'s investigations[1]. For $CoSb_3$, it is found that the IFCs almost vanish at a cutoff interatomic distance of 6.5 Å. Therefore, a conventional unit cell with a lattice constant of ~ 9 Å can provide reliable calculation results for $CoSb_3$. Note that the long-ranged and non-monotonically decreasing interactions are due to the long-ranged electronic polarizability, which are different from the long-ranged Coulomb interaction[1].

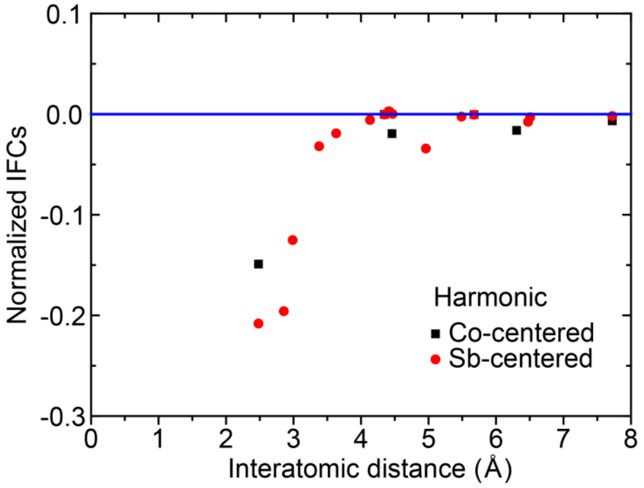

Supplementary Figure 1 | Normalized harmonic IFCs versus interatomic distance for $CoSb_3$. The center atom indicates the interaction between the corresponding atom and other atoms. For example, "Sb-centered" means the interaction between Sb and other atoms in $CoSb_3$.

References

1. Lee, S. *et al*. Resonant bonding leads to low lattice thermal conductivity. *Nat. Commun.* **5**, 3525 (2014).
